# Supplementary material for: MetaRibo-Seq measures translation in microbiomes
Source: Nat Commun. 2020 Jun 29;11:3268. doi: 10.1038/s41467-020-17081-z (PMC7324362; doi:10.1038/s41467-020-17081-z)
Supplement: Supplementary file 10 — Supplementary Data 7 [file 41467_2020_17081_MOESM10_ESM.zip › File2/Confidence_High_Taxonomy/83866_out.krona.html]

Javascript must be enabled to view this page.

members
magnitude
magnitudeUnassigned
count
unassigned
taxon
rank

83866\_out

26

superkingdom
2
26

phylum
976
26

26
200643
class

order
26
171549

171552
26
family

genus
838
26


SRS012849\_contig\_number\_5746SRS014415\_contig\_number\_2007SRS015782\_contig\_number\_40099SRS015794\_contig\_number\_12247SRS017307\_contig\_number\_19820SRS018888\_contig\_number\_9458SRS019285\_contig\_number\_contig-100\_6.7SRS024132\_contig\_number\_23623SRS044535\_contig\_number\_12591SRS046502\_contig\_number\_13647SRS047044\_contig\_number\_12622SRS049712\_contig\_number\_2884SRS049959\_contig\_number\_2770SRS050752\_contig\_number\_264SRS065397\_contig\_number\_9892SRS074586\_contig\_number\_contig-100\_219.55193SRS077194\_contig\_number\_11016SRS077641\_contig\_number\_21638SRS078419\_contig\_number\_11677SRS104036\_contig\_number\_7971SRS105082\_contig\_number\_355SRS143181\_contig\_number\_2165SRS143876\_contig\_number\_3049SRS144506\_contig\_number\_57224SRS146764\_contig\_number\_25171SRS147614\_contig\_number\_7572
165179
26
species
